# Potency of irritation by benzylidenemalononitriles in humans correlates with TRPA1 ion channel activation

**DOI:** 10.1098/rsos.140160

**Published:** 2015-01-28

**Authors:** Christopher D. Lindsay, Christopher Green, Mike Bird, James T. A. Jones, James R. Riches, Katherine K. McKee, Mark S. Sandford, Debra A. Wakefield, Christopher M. Timperley

**Affiliations:** Defence Science and Technology Laboratory (Dstl), Porton Down, Salisbury, Wiltshire SP4 0JQ, UK

**Keywords:** 2-chlorobenzylidenemalononitrile, tear gas, TRPA1, benzylidenemalononitriles, irritancy, riot control agent

## Abstract

We show that the physiological activity of solid aerosolized benzylidenemalononitriles (BMNs) including ‘tear gas’ (CS) in historic human volunteer trials correlates with activation of the human transient receptor potential ankyrin 1 ion channel (hTRPA1). This suggests that the irritation caused by the most potent of these compounds results from activation of this channel. We prepared 50 BMNs and measured their hTRPA1 agonist potencies. A mechanism of action consistent with their physiological activity, involving their dissolution in water on contaminated body surfaces, cell membrane penetration and reversible thiolation by a cysteine residue of hTRPA1, supported by data from nuclear magnetic resonance experiments with a model thiol, explains the structure–activity relationships. The correlation provides evidence that hTRPA1 is a receptor for irritants on nociceptive neurons involved in pain perception; thus, its activation in the eye, nose, mouth and skin would explain the symptoms of lachrymation, sneezing, coughing and stinging, respectively. The structure–activity results and the use of the BMNs as pharmacological tools in future by other researchers may contribute to a better understanding of the TRPA1 channel in humans (and other animals) and help facilitate the discovery of treatments for human diseases involving this receptor.

## 2. Introduction

**Figure 1.**
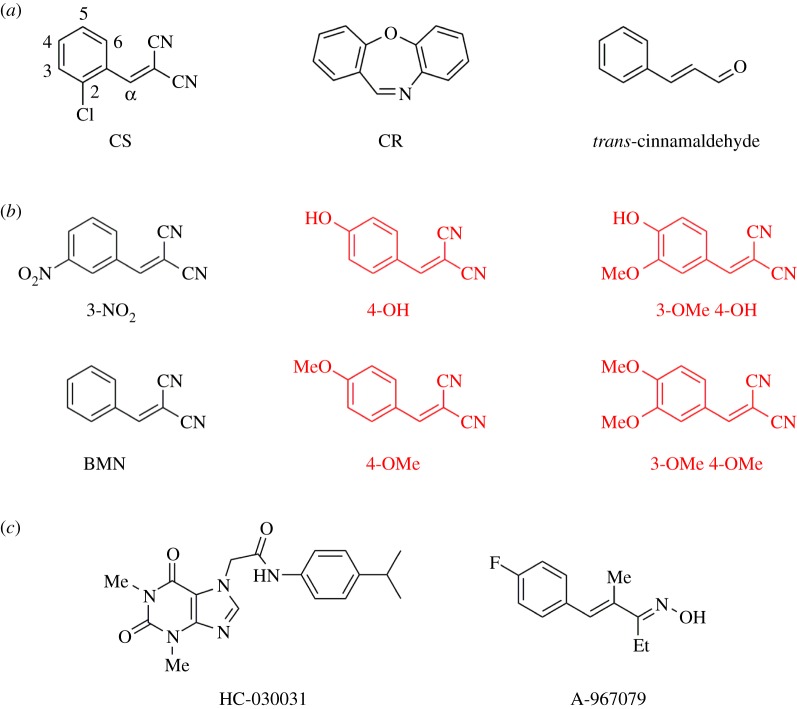
Compounds of interest. (*a*) Some hTRPA1 agonists. Numbering used to abbreviate the substituted CS analogues is shown. (*b*) Structure–irritancy relationships found by early workers [1,2]: compounds in black are irritant, those in red are inactive. In 1928, Corson & Stoughton [2] noted that some BMNs had the ‘effect of sneeze and tear gases’. When crystallizing 3-nitrobenzylidenemalononitrile (3-NO_2_), they found that the ‘alcohol solution should not be boiled very much as the alcohol vapour has a peppery sting’. In sneezing caused by this analogue the mucous discharge from the nose became ‘bright yellow on exposure to air’. The irritant action of CS was noted as ‘sneeze and skin’ and BMN as ‘sneeze and tear’. (*c*) Some hTRPA1 antagonists that inhibit channel activation by CS.

The irritancy of BMNs was recognized 100 years ago by Heller and Wunderlich who found that 2-nitrobenzylidenemalononitrile ‘strongly irritates the mucous membranes’ [1]. In 1928, Corson and Stoughton noted that 2-chlorobenzylidenemalononitrile (CS) and its 3-NO_2_ analogue (figure 1*a*,*b*) acted similarly [2]. CS provoked sneezing and caused smarting of the face, especially when damp. This sensation intensified on washing. The parent compound BMN was also irritant and the 4-hydroxy/methoxy-BMNs non-irritant (figure 1*b*).

In the 1930s, scientists at the Chemical Defence Experimental Station, Porton Down [3], examined the physiological activity of CS, its 2-NO_2_ and 2-Br analogues, and BMN by exposing human volunteers to aerosols in a chamber. The compounds caused powerful irritation, and other analogues were prepared to try to determine the cause of their physiological activity. This was not found but research continued at a low priority until 1958 whereupon a safe riot control agent was sought. This prompted a reassessment of a greater range of compounds leading to the selection of CS [4]. This time, rather than resorting immediately to chamber trials, BMNs of unknown physiological activity were first subjected to a rough laboratory screening test by vaporizing a small quantity on a hotplate and cautiously exposing the eyes and nasal airway to the condensation aerosol. In this way, it was possible to eliminate the less promising compounds. The remainder were then more fully tested by exposing human volunteers to known concentrations in the chamber. From these studies, and other accounts of exposures of human subjects to CS [5–10], it was possible to reach a number of conclusions, such as the maximum activity had probably been reached with CS and some simple derivatives. A substituent in the 2-position of the ring greatly increased physiological activity, a 3-substituent lessened the effect and a 4-substituent reduced the activity ‘frequently to vanishing point’ [11]. Further, for pronounced activity, the double bond was essential and also the two cyanide groups, although some activity still persisted if one (or both) of the latter was replaced by a strongly electronegative group.

Despite these studies, an understanding of how the stereoelectronic properties of BMNs influenced their biological activity remained out of reach. The statement made in 1936 by Porton scientists that, ‘on account of the almost total lack of biochemical data in this field, no attempt has been made to suggest a mode of action for these compounds’ [12], remained pertinent until 2008. Then the biological target for CS [13,14], the riot control agent CR [13–15] and the cinnamon constituent *trans*-cinnamaldehyde (figure 1*a*) was identified as the human transient receptor potential ankyrin 1 (hTRPA1) ion channel [16], a non-selective calcium permeable channel. When activated, the channel permits the movement of cations (primarily Ca^2+^ and Na^+^) from the extracellular environment into the cell, thus depolarizing the membrane potential and affecting calcium homeostasis in primary afferent neurons. Their depolarization leads to action potential firing and consequently increased pain sensation. The hTRPA1 ion channel is present in neurons of the dorsal root ganglia (DRG) and trigeminal ganglia of the sensory primary afferents [17]. The sensitive surfaces of the eyes, olfactory and respiratory intranasal mucosa are innervated by free nerve endings of the trigeminal nerve [18]. Information from trigeminal stimulation by chemical irritants is transmitted and processed into the sensation of pain in the brain [19,20].

CS, CR and *trans*-cinnamaldehyde are postulated to activate hTRPA1 by reversible covalent reaction with a cysteine thiol group [13–15,21,22] in the *N*-terminal ankyrin repeat domain of this membrane-bound protein [23], presumably causing conformational changes that cause the channel to open [24]. CS and CR are potent agonists of hTRPA1, having EC_50_ values in the nanomolar/subnanomolar range [13–15]. Genetic mutations in ion channels are implicated in a diverse range of pathological conditions that regulate pain [25], and the importance of hTRPA1 in the physiology of pain has been reinforced by the finding that a gain-of-function mutation in humans confers an episodic pain syndrome [26]. Human TRPA1 has been detected in DRG neurons, spinal cord motor neurons, nerve roots and peripheral nerves [27]. *trans*-Cinnamaldehyde evokes coughing in humans [28,29] and has a burning taste [30] like CS [31] and CR [32]. Several TRPA1 antagonists (figure 1*c*) [33] are able to modulate the function of the ion channel and counteract the activation caused by *trans*-cinnamaldehyde and CS [34–36].

Here, we report the synthesis of a series of 50 BMNs and measurement of their ability to activate hTRPA1 in human embryo kidney (HEK) 293 cells by Ca^2+^ fluorimetry. The assay results were compared with irritancy data from human volunteer trials that were conducted at the Chemical Defence Experimental Establishment, Porton Down, in the 1950s [4]. We propose a chemical mechanism explaining hTRPA1 activation by the BMNs that rationalizes all the structure–irritancy relationships currently available. This study is the first to correlate the severity of irritancy to humans from a family of aerosolized chemicals to a mechanism and supports the function of TRPA1 as a sensor for chemically induced irritation, which should help biomedical research into strategies for treating hTRPA1-mediated pain.

## 3. Material and methods

### 3.1 Materials

(Pentafluorosulfur)benzaldehydes were purchased from JRD Fluorochemicals Ltd. (Surrey, UK) and other aldehydes from Aldrich Ltd. (Dorset, UK). Pure CR was synthesized in-house according to a literature method [37]. Reagents for the hTRPA1 assay were from Sigma-Aldrich Ltd. unless stated. G418 was from Calbiochem (Merck KGaA, Darmstadt, Germany) and Gibco Hank's balanced salts solution (HBSS) from Life Technologies Ltd. (Paisley, UK). The HEK Photoscreen hTRPA1 cell line (AX-004-PCL) was obtained from PerkinElmer Life and Analytical Sciences (Waltham, MA, USA).

### 3.2 Analysis

Nuclear magnetic resonance (NMR) data were collected at 9.4 or 14.1 T by a Bruker AVIII 400 or 600 MHz spectrometer, respectively. The AVIII 400 data were collected using a 5-mm BBFO+ probe. The AVIII 600 was equipped with a ^1^H/^31^P/^13^C/^15^N/D 5 mm QCI-P cryoprobe. Gas chromatography–mass spectrometry (GC–MS) in electron ionization mode (70 eV) was performed on dichloromethane solutions of BMNs on an Agilent Technologies 6890 GC interfaced to a 5973 mass selective detector from the same manufacturer (Cheshire, UK). The average linear retention index (LRI) was recorded (*n*=3) using the 25 m Agilent Technologies J&W DB-5 MS column (0.2 mm internal diameter, 0.33 μm film thickness) by reference to an alkane test mix (C7–C30) with the initial temperature set to 40°C (hold 5 min) and programmed to rise to 280°C at 10°C min^−1^ (hold 15 min). Helium carrier gas was used in constant flow mode (1 ml min^−1^). A 1-μl splitless injection was performed at 280°C. Mass-to-charge ratios (*m*/*z*) of the 10 most abundant ions (% abundance) are listed.

### 3.3 Synthesis

All the compounds reported in this paper were synthesized specifically for the sole purpose of seeing if their agonist potency measured by the hTRPA1 channel assay related to their irritancy determined in the historic chamber trials. None of the compounds originated from archived collections; those tested in the trials were destroyed long ago. The trials predated the advent of modern analytical techniques such as NMR spectroscopy and mass spectrometry. For the early Porton researchers, repeated crystallization of each BMN to a constant melting point, sometimes assisted by reference literature data, and satisfactory microanalytical data, were used as criteria of high purity. In the compound listings that follow in this section and in the electronic supplementary material, the melting points obtained for the resynthesized BMNs are referenced alongside those recorded in the original journal articles or Porton papers. For the hTRPA1 screen, compounds of 98% purity, as determined by ^1^H NMR spectroscopy, were used and their melting points generally agreed closely with those reported for samples used in the historic trials, suggesting a comparable state of purity.

We used two methods to prepare the BMNs: (Method A) The aldehyde (40 mmol) was added to a stirred mixture of malononitrile (2.64 g, 40 mmol) and 1-methylimidazole (0.03 g, 0.4 mmol) in water (21.6 ml) at 35°C [38]. Crystalline product formed typically within 30 min and was filtered off and dried under vacuum; (Method B) the aldehyde (20 mmol), malononitrile (1.32 g, 20 mmol) and isopropanol (15 ml) were stirred magnetically in a 50-ml round-bottomed flask [39]. Full solution was achieved by gentle warming. A few drops of piperidine were added once the solution had cooled to room temperature. Solid crystalline product usually formed immediately. It was filtered off and dried under vacuum.

*2-Methylbenzylidenemalononitrile* (*2-Me*). Method A. Fine cream crystals (97%). Mp 106°C. ^1^H NMR (400 MHz, CDCl_3_) 8.11–8.07 (2H, m), 7.50 (1H, dd, *J*=7.3, 7.3 Hz), 7.39–7.31 (2H, m), 2.45 (3H, s). ^13^C{^1^H} NMR (100 MHz, CDCl_3_) 158, 139, 134, 131, 129, 128, 127, 113, 112, 84, 19. LRI 1530, *m*/*z* (%): 168 (36), 142 (12), 141 (100), 140 (56), 114 (34), 113 (13), 89 (12), 63 (18), 51 (15), 50 (12).

*2-Ethylbenzylidenemalononitrile* (*2-Et*). Method B. Rinsed with isopropanol then petroleum ether (bp 40–60°C) to give white crystals (54%). Mp 63–64°C. ^1^H NMR (600 MHz, CDCl_3_) 8.14 (1H, s), 8.07 (1H, d, *J*=7.9 Hz), 7.53 (1H, dd, *J*=7.3, 7.3 Hz), 7.38–7.34 (2H, m), 2.76 (2H, q, *J*=7.6 Hz), 1.24 (3H, t, *J*=7.6 Hz). ^13^C{^1^H} NMR (150 MHz, CDCl_3_) 158, 146, 134, 129.8, 129, 128.5, 127, 113, 112, 84, 26, 16. LRI 1583, *m*/*z* (%): 182 (56), 181 (76), 167 (17), 155 (17), 154 (28), 141 (19), 140 (100), 127 (15), 115 (23), 63 (12).

2-(*Pentafluorosulfanyl*)*benzylidenemalononitrile* (*2*-*SF*_5_). Method A. Pale yellow solid (82%). Mp 84°C. ^1^H NMR (400 MHz, CDCl_3_) 8.19–8.15 (2H, m), 8.03–7.99 (1H, m), 7.84 (1H, s), 7.70 (1H, dd, *J*=8.2, 8.2 Hz). ^13^C{^1^H} NMR (100 MHz, CDCl_3_) 157, 154, 132, 131.4, 131.1, 130, 128, 112, 111, 86. ^19^F{^1^H} NMR (376 MHz, CDCl_3_) 81 (1F, quint, ^2^*J*_FF_=148 Hz), 62 (4F, d, ^2^*J*_FF_=150 Hz). LRI 1598, *m*/*z* (%): 280 (70), 172 (75), 153 (68), 145 (100), 127 (34), 126 (61), 121 (59), 89 (38), 75 (33), 100 (32).

3-(*Pentafluorosulfanyl*)*benzylidenemalononitrile* (*3*-*SF*_5_). Method A. Recrystallized from hot ethanol to yield fine pale cream needles. Mp 88°C. ^1^H NMR (400 MHz, CDCl_3_) 8.22–8.17 (2H, m), 8.05–8.02 (1H, m), 7.87 (1H, s), 7.73 (1H, dd, *J*=8.0, 8.0, 8.2 Hz). ^13^C{^1^H} NMR (100 MHz, CDCl_3_) 157, 154, 132, 131.4, 131.1, 130, 128, 112, 111, 86. ^19^F{^1^H} NMR (376 MHz, CDCl_3_) 81 (1F, quint, ^2^*J*_FF_=152 Hz), 62 (4F, d, ^2^*J*_FF_=150 Hz). LRI 1601, *m*/*z* (%): 280 (70), 172 (44), 153 (92), 145 (100), 127 (39), 126 (79), 121 (59), 100 (34), 89 (46), 75 (34).

4-(*Pentafluorosulfanyl*)*benzylidenemalononitrile*(*4*-*SF*_5_). Method A. Recrystallized from hot ethanol to yield pale yellow needles. Mp 128°C. ^1^H NMR (400 MHz, CDCl_3_) 8.00 (2H, d, *J*=8.7 Hz), 7.94 (2H, d, *J*=9.0 Hz), 7.84 (1H, s). ^13^C{^1^H} NMR (100 MHz, CDCl_3_) 157.2, 157.1, 133, 130, 127, 112, 111, 86. ^19^F{^1^H} NMR (376 MHz, CDCl_3_) 81 (1F, quint, ^2^*J*_FF_=149 Hz), 62 (4F, d, ^2^*J*_FF_=150 Hz). LRI 1610, *m*/*z* (%): 280 (26), 172 (100), 153 (31), 145 (61), 126 (26), 121 (37), 100 (15), 99 (16), 89 (18), 75 (17).

Data for the other 45 BMNs prepared in this study appear in the electronic supplementary material.

### 3.4 Cell culture

HEK hTRPA1 cells were grown in 150 cm^2^ flasks at 37°C (5% CO_2_–95% air) (Corning Inc., NY, USA) in culture medium (Earle's modification of Eagle's medium (EMEM) supplemented with fetal bovine serum (10% v/v); penicillin and streptomycin (100 units ml^−1^ and 100 μg ml^−1^, respectively), l-glutamine (2 mmol) and G418 (0.4 mg ml^−1^)). At 80–90% confluency, the cells were passaged at split ratios of 1:3 to 1:16 for continued growth in new flasks, or for sowing into poly-d-lysine (PDL)-coated 96-well black Biocoat cell culture plates (BD Biosciences, Bedford, MA, USA) for Ca^2+^ assays. Cells were washed with Ca^2+^- and Mg^2+^-free Dulbecco's phosphate buffered saline (DPBS, 12 ml) and detached using a solution of trypsin/EDTA(Na)_4_ in DPBS for 5 min at 37°C. Detached cells were collected by centrifugation (80*g* for 4 min at 20°C) and the resulting pellet was resuspended in culture medium. Cells were counted and cell viability determined using trypan blue. The cells were sown into PDL-coated plates at a density of 12 500 viable cells per well and were grown for 48 h prior to use. Cells at passage numbers 10–22 were used.

### 3.5 hTRPA1 agonism

HEK hTRPA1 cells were loaded with a Ca^2+^ fluorophore using a Calcium 5 assay kit (Molecular Devices Ltd., Berkshire, UK). Loading buffer containing the Ca^2+^ fluorophore was prepared by supplementing every 10 ml of the proprietary loading buffer with assay buffer (5 ml) comprising a 1:1 v/v mixture of EMEM and HBSS (buffered to pH 7.4 with 20 mmol HEPES). Cells in 96-well plates were loaded in preparation for the assay by removal of the EMEM and addition of the loading buffer (90 μl) to each well. The cells were then incubated for 30 min at 37°C in a humidified atmosphere of 5% CO_2_–95% air. The BMNs were dissolved in dimethyl sulfoxide (DMSO) prior to being diluted in assay buffer to a concentration of 4 mM. Subsequently, serial dilutions of these stocks were prepared in 96-well plates (Costar brand, Corning Inc.) to provide a semi-log dilution series from 0 to 12 μmol. *trans*-Cinnamaldehyde (Aldrich Ltd.) was similarly treated except that the initial stock was made up in assay buffer only.

Cellular fluorescence was measured at 1 s intervals before and after addition of the BMN solution (30 μl) using a FlexStation 3 (Molecular Devices Ltd., Sunnyvale, CA, USA). This resulted in the cells being exposed to final BMN concentrations from 0 to 3 μM in a semi-log progression. Fluorescence from formation of the Calcium-5−Ca^2+^ complex was measured using excitation wavelength 485 nm and emission wavelength 525 nm. Baseline fluorescence data were collected for 15 s prior to addition of the BMN solution and the response followed for a further 45 s. The final concentration of DMSO in these assays was always less than 1.9×10^−5^% v/v and control experiments showed no effect from the DMSO at this concentration.

### 3.6 hTRPA1 antagonism

HC-030031 and A-967079 concentration–effect relationships were determined by modifying the methods already described. HEK hTRPA1 cells in 96-well plates were loaded in loading buffer (60 μl) supplemented with the required antagonist dilution (30 μl) in assay buffer. Initial solutions of antagonists were made up in DMSO prior to dilution with assay buffer. Cells were incubated for 30 min at 37°C in a humidified atmosphere of 5% CO_2_–95% air prior to challenge with a fixed concentration of CS (100 nM) and the cellular calcium response recorded as described. The final concentration of DMSO was always less than 0.063% v/v and control experiments showed no effect from the DMSO at this concentration.

### 3.7 Data analysis

Fluorescence emission data (fluorescence intensity versus time) were captured from the FlexStation 3 using SoftMax Pro software v. 5.4.1 (Molecular Devices Inc., 1992–2010) on a Dell Optiplex 960 PC workstation. The area under the time–response curve was calculated for the entire recording and imported into GraphPad Prism v. 4.03 (GraphPad Software Inc., San Diego, CA, USA) for nonlinear curve fitting. Raw concentration–response data were fitted with a four parameter logistic equation: *Y* =Bottom+(Top−Bottom)/(1+10((LogEC_50_−*X*)×Slope), where *X*=logarithm of concentration and *Y* =response. The EC_50_ and 95% confidence interval (CI) were obtained. Maximum and minimum fluorescence responses were normalized to 100% and 0%, respectively, to permit comparison of hTRPA1 agonist activity of the test compounds and controls, and presentation of the concentration–response data. For BMNs which failed to elicit significant responses, a maximum response equal to that achieved by potent agonists in the same experiment was assumed in order to display these data on the normalized graphs. Each data point represents the mean value from at least five independent experiments ±s.e.m.

### 3.8 Volunteer trials

Currently, the UK Ministry of Defence (MoD) Research Ethics Committees ensure that all research involving human participants undertaken, funded or sponsored by the MoD meets UK and internationally accepted ethical standards [40]. The trials on sensory irritants reported herein were conducted over 50 years ago as part of the Porton Down Service Volunteer Programme after gaining what at the time was regarded as the appropriate approval. Sir Ian Kennedy's independent assessment of human participation in research at Porton Down, commissioned by the MoD as part of the Historical Survey of the Porton Down Service Volunteer Programme [41], was critical of a small number of trials conducted around this time, all of which involved exposures to nerve agents. However, Sir Ian also concluded that for the majority of trials, ‘research was carried out at Porton in a thorough, painstaking, careful and often ingenious manner. Examples include … the elaborate and extensive trials of riot control agents (not least because the safety of the wider public was at stake)’ and that with respect to these trials, ‘there is no evidence to justify a conclusion that the conduct of the trials at any point went beyond the limits of what should ever be contemplated, far less tolerated, in a civilized society’.

In the trials [3,4,42–44], a known weight of the test compound was dissolved in an organic solvent (15 ml) and the solution transferred into a ‘sprayer of special design’ (no further details available) [45]. The solution was sprayed into the chamber by compressed air. A ‘few ml of the solvent’ was added to rinse the apparatus and ensure that all the solute had been dispersed. As BMNs are solids with very low vapour pressures at room temperature, their physical state when dispersed in the chamber was that of a particulate cloud. The physiological response to such clouds depends upon the particle size, which may change with the age of the cloud. Large particles are caught in the nose by sedimentation or impingement; 10 μm particles are mostly caught in the bronchi, and 3 μm particles in the bronchioles; to reach the alveoli, the particles must be around 1 μm in diameter. The effect of particle size of CS aerosol on irritation in humans depends on several factors. Larger particles (60 μm) cause predominantly ocular irritation [6]. Smaller particles (0.9 μm) rapidly produce respiratory and ocular irritation. Recovery from eye irritation from smaller particles takes longer. One explanation proposed to account for the difference in severity of ocular irritation from large and small particles is that the latter, although able to impact the eye less efficiently, dissolve faster in eye fluid [6]. However, with the smaller particles diffusion will be important, not impaction, particularly at low air speeds, which is why they are so efficient relative to the large particles. In the volunteer trials at Porton Down [3,4,42–44], classification of the action of a compound as sternutatory or lachrymatory was recorded by the subjects, and often both actions were observed.

### 3.9 Model thiol experiments

Solutions of the irritant (1 M) and *N*-acetyl-l-cysteine methyl ester (1 M) in CDCl_3_ were prepared. The irritant solution (300 μl) was added to the thiol solution (300 μl) in a 5 mm NMR tube. Kinetic measurements were recorded at 14.1 T using the Bruker AVIII 600. The temperature was regulated using a standard broker BVT system. Spectra were recorded at 25°C after mixing (time from start *t*=0 min) and later (*t*=2 h). The temperature was raised to 37°C and spectra recorded 2 and 4 h later (*t*=4 h and 6 h). The temperature was returned to 25°C and spectra recorded after 4 h (*t*=10 h) and 14 h (*t*=24 h). The percentage of product was calculated at each interval by integrating the relevant signals.

## 4. Results and discussion

### 4.1 Action of benzylidenemalononitriles on the hTRPA1 ion channel

We synthesized and characterized 50 BMNs with groups at different positions on the ring. They included some of those tested historically on humans and more than 20 new analogues. Perhaps the most interesting of the latter were the ones containing the uncommon and biologically stable difluoromethoxy (OCF_2_H) and pentafluorosulfur (SF_5_) substituents; the first BMNs containing these substituents are reported herein. We measured the concentration of the BMNs eliciting a half maximal increase of the Ca^2+^ response (EC_50_) and 95% CI using the hTRPA1 assay (table 1). CR, CS and *trans*-cinnamaldehyde were used as positive controls as their agonist potencies were known [13–15].

**Table 1.** Measured hTRPA1 responses for BMNs in order of potency (most potent at top). All determinations were from five independent experiments except those denoted by a, b and c.

analogue	2-	3-	4-	5-	6-	log_10_ EC_50_ (M)	95% CI
CR	—	—	—	—	—	−8.743^a^	−8.820 to −8.666
2-Cl (CS)	Cl	H	H	H	H	−8.102^b^	−8.156 to −8.047
2-NO_2_	NO_2_	H	H	H	H	−8.065	−8.133 to −7.997
2-F	F	H	H	H	H	−8.022	−8.150 to −7.895
2-OCF_2_H	OCF_2_H	H	H	H	H	−7.987	−8.083 to −7.890
2-Br	Br	H	H	H	H	−7.979	−8.091 to −7.867
2-CF_3_	CF_3_	H	H	H	H	−7.969	−8.063 to −7.876
2,6-di-Cl	Cl	H	H	H	Cl	−7.884	−7.985 to −7.784
2,3,6-tri-Cl	Cl	Cl	H	H	Cl	−7.818	−7.905 to −7.731
2-I	I	H	H	H	H	−7.791	−7.860 to −7.722
2-Cl-3-OH	Cl	OH	H	H	H	−7.472	−7.552 to −7.392
2-Me	CH_3_	H	H	H	H	−7.370	−7.453 to −7.287
2,4-di-Cl	Cl	H	Cl	H	H	−7.248	−7.315 to −7.180
3-F	H	F	H	H	H	−7.214	−7.290 to −7.138
3-I	H	I	H	H	H	−7.179	−7.407 to −6.950
3-OH	H	OH	H	H	H	−7.175	−7.218 to −7.132
3-OMe	H	OMe	H	H	H	−7.135	−7.229 to −7.041
2,6-di-F	F	H	H	H	F	−6.950	−7.110 to −6.790
3-Br	H	Br	H	H	H	−6.943	−7.064 to −6.823
3-CF_3_	H	CF_3_	H	H	H	−6.869	−6.978 to −6.761
BMN	H	H	H	H	H	−6.847	−6.905 to −6.788
3-Cl	H	Cl	H	H	H	−6.836	−6.884 to −6.787
2,3-di-Cl	Cl	Cl	H	H	H	−6.807	−6.967 to −6.646
3-SF_5_	H	SF_5_	H	H	H	−6.760	−6.927 to −6.594
2-SF_5_	SF_5_	H	H	H	H	−6.677	−6.860 to −6.493
2-Et	Et	H	H	H	H	−6.533	−6.575 to −6.491
2-OMe	OMe	H	H	H	H	−6.531	−6.606 to −6.456
cinnamaldehyde^c^	—	—	—	—	—	−4.477	−4.522 to −4.431
1-naphthyl	—	—	—	—	—	92.6^d^	—
2-CN	CN	H	H	H	H	88.8^d^	—
4-F	H	H	F	H	H	88.6^d^	—
2,3,6-tri-F	F	F	H	H	F	88.4^d^	—
3-CN	H	CN	H	H	H	87.5^d^	—
2-F-5-SF_5_	F	H	H	SF_5_	H	86.8^d^	—
2,5-di-Cl	Cl	H	H	Cl	H	86.7^d^	—
4-Br	H	H	Br	H	H	86.6^d^	—
4-I	H	H	I	H	H	84.3^d^	—
3,4-di-Cl	H	Cl	Cl	H	H	75.5^d^	—
2-F-4-SF_5_	F	H	SF_5_	H	H	75.2^d^	—
3-NO_2_	H	NO_2_	H	H	H	72.7^d^	—
2-OEt	OEt	H	H	H	H	71.1^d^	—
4-Cl	H	H	Cl	H	H	67.4^d^	—
3,5-di-Cl	H	Cl	H	Cl	H	51.6^d^	—
4-CF_3_	H	H	CF_3_	H	H	42.5^d^	—
4-SF_5_	H	H	SF_5_	H	H	38.4^d^	—
3-OMe-4-OH	H	OMe	OH	H	H	17.3^d^	—
3,4-di-OEt	H	OEt	OEt	H	H	10.5^d^	—
4-NO_2_	H	H	NO_2_	H	H	9.7^d^	—
4-CN	H	H	CN	H	H	9.6^d^	—
3,4-di-OMe	H	OMe	OMe	H	H	9.2^d^	—
4-OMe	H	H	OMe	H	H	8.5^d^	—
4-OH	H	H	OH	H	H	1.6^d^	—

^a^*n* = 10.

^b^*n* = 20.

^c^*trans*-isomer, *n* = 20.

^d^EC_50_ could not be adequately determined by fitting the raw data; data presented as % of maximum mean fluorescence at 3 μM, as titration curves did not reach completion.

**Figure 2.**
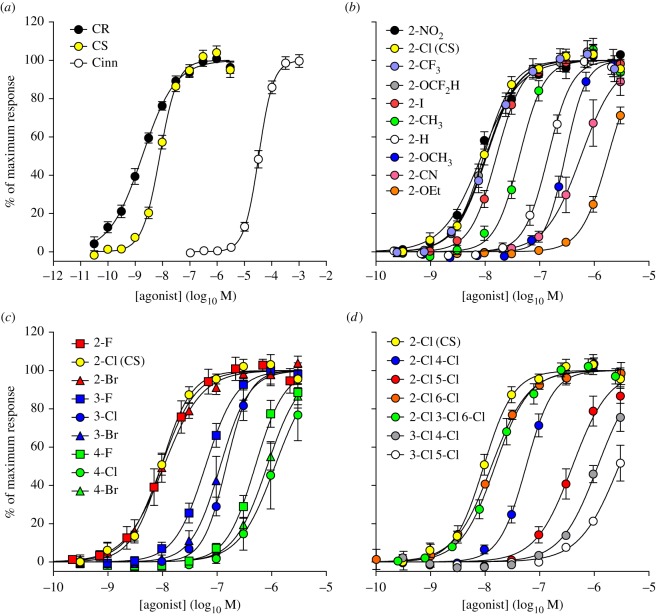
Relationship of structure to hTRPA1 activation. (*a*) Activation of hTRPA1 by CR, CS and *trans*-cinnamaldehyde (Cinn). (*b*) Concentration–response curves for 2-substituted BMNs; the 2-NO_2_ analogue (black) has potency similar to CS (yellow). (*c*) Concentration–response curves for 2-halogen substituted BMNs showing the potency order: 2-substituted (red) > 3-substituted (blue) > 4-substituted (green). (D) Concentration–response curves for polychlorinated BMNs showing that multiple chlorine atoms reduce agonist potency. The most potent analogues contain a chlorine atom in the 2- (or 6-) position: CS (yellow), 2-Cl 6-Cl (orange) and 2-Cl 3-Cl 6-Cl (green).

Agonist potency declined in the order CR > CS ≫ *trans*-cinnamaldehyde (figure 2*a*), as expected [13], reflecting their relative irritancy to humans (but not as aerosols) [31,32]. The observation that 2-substituted BMNs are more irritating than 3-substituted BMNs, and far more irritating than 4-substituted BMNs, mirrored the EC_50_ values found. Values for the 2-substituted BMNs decreased as follows: CS (2-Cl) > 2-NO_2_ > 2-F > 2-OCF_2_H > 2-Br > 2-CF_3_ > 2-I > 2-Me > 2-H (BMN) > 2-SF_5_ > 2-Et > 2-OMe > 2-CN > 2-OEt (figure 2*b*). For compounds with the same group in different positions of the ring, potency fell in the order 2- > 3- > 4-substituted (figure 2*c*). Extra chlorine atoms on the ring reduce irritancy to humans [46] and EC_50_ values decreased accordingly (figure 2D). HC-030031 and A-967079 blocked the CS response (electronic supplementary material, figure), with potencies consistent with those reported elsewhere [47,48], confirming that the response was mediated by hTRPA1 receptors.

### 4.2 Irritancy correlates with hTRPA1 activation

In the trials conducted at Porton Down in the 1930s, human volunteers were exposed in a 100 m^3^ chamber to concentrations of BMNs dispersed in benzene (2-NO_2_, CS, BMN) or acetone (2-Br) [3,42–44]. The tests of the 2-NO_2_ analogue and CS (table 2) confirmed the observations of Heller & Wunderlich [1] and Corson & Stoughton [2]. Volunteers exposed to 0.01 ppm reached the limit of their tolerance within 1 min and rapidly left the chamber. The compounds were subsequently assessed as lachrymatory or sternutatory—irritating the eye or respiratory tract—to a ‘degree sufficient to render work impossible’ [3]. From these data, the physiological activity appeared to decline in the order CS (2-Cl) ∼ 2-NO_2_ > 2-Br ≫ BMN (2-H), which correlates with the EC_50_ values for these compounds measured in the hTRPA1 assay (figure 2*b*). All four irritated the eyes and respiratory tract, but only the first three caused ‘prickling’ of the skin at the concentrations studied.

**Table 2.** Physiological action on male human volunteers of four BMNs dispersed in a 100 m^3^ chamber by spraying in benzene or acetone^a^.

compound^b^	concentration	physiological symptoms	conclusion
2-Cl (CS) [3] 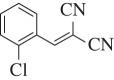	0.01 ppm (benzene)	detected immediately by ‘peppery’ irritation of the upper respiratory passages, the throat and chest were involved within 15 s, and lachrymation rapidly followed. ‘Prickling’ effect on skin was noted. This concentration was considered about the limit of tolerability for 1 min exposure	2-Cl is a lachrymator and sternutator. It is immediately intolerable in a concentration of 0.2 ppm, and in a concentration as low as 0.01 ppm causes eye irritation, lachrymation and respiratory irritation sufficient to render work impossible if there is no protection. The irritant symptoms subside rapidly on leaving the chamber
	0.2 ppm (benzene)	this concentration was immediately intolerable by reason of ‘peppery’ irritation of the whole respiratory tract, profuse lachrymation and ‘prickling’ effect on exposed skin	
2-NO_2_[42] 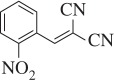	0.01 ppm (benzene)	immediate and severe irritation of the whole respiratory tract with much coughing followed by ‘prickling’ of the skin particularly where it was moist, and irritation of the eyes with lachrymation. The limit of tolerability was reached in 35 s and subjects left the chamber	2-NO_2_ is a powerful sternutator and lachrymator at a nominal concentration of 0.01 ppm. At a nominal concentration of 0.002 ppm, it produces physiological symptoms which, though not intolerable, would seriously interfere with the performance of duties. Prickling of the skin, particularly where it is moist is a notable symptom. The physiological symptoms rapidly subside after leaving the experimental atmosphere
	0.002 ppm (benzene)	the whole respiratory tract was involved within 1 min. The irritation which was of the ‘hot peppery’ type induced coughing and a desire to sneeze. Eye irritation developed and also slight ‘prickling’ of the skin. The exposure was limited to 10 min and though not intolerable, this concentration was decidedly uncomfortable and would seriously interfere with work. The eye irritation did not progress to lachrymation	
2-Br [43] 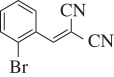	0.01 ppm (acetone)	rapid onset of irritation of the eyes, nose, throat and in some cases the chest all within 15 s. Three out of five subjects left the chamber in 60 s, 90 s and 210 s, respectively. The other two subjects remained in for 10 min and reported that the symptoms tended to subside during exposure. Throat and chest irritation was not marked. Prickling of the skin, particularly where moist, was noted	2-Br possesses lachrymatory and sternutatory properties. In addition, ‘prickling’ of the skin particularly over moist areas is a feature with this compound. All of the symptoms rapidly subsided after leaving the chamber
	0.2 ppm (acetone) (men equipped with respirators)	the object of this exposure was to observe the irritant effects on exposed skin in a higher concentration. Protected with gas mask and with the exposed skin at normal (cool) temperature, ‘prickling’ was not marked and somewhat slow in developing. One subject removed his respirator and found the concentration quite irrespirable. This was followed by marked prickling of the skin on the moist areas of face, and on lachrymation marked stinging of the skin along the course of tears on the face	
2-H (BMN) [44] 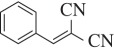	0.2 ppm (benzene)	detected immediately by irritation of the upper respiratory passages. The irritation was of the ‘peppery’ type. The chest became affected within 1 min causing mild coughing and restricted breathing. There was also tear formation but no actual lachrymation. Although this concentration would interfere with carrying out ordinary duties, the symptoms could not be described as indicating the limit of tolerability during an exposure of 3 min. Towards the end of this exposure, the discomfort was subsiding	BMN is an irritant which attacks the respiratory tract and to a lesser degree the eyes. In both respects, however, it is far less potent than many other well-known compounds

^a^The wording in the table is taken without alteration from Porton Down Reports [3,42–44].

^b^The BMNs used in the historic study summarized in this table were prepared by the method in the original paper of Corson and Stoughton (from whose initials CS derives its codename) [2]. The 2-NO_2_ and 2-Br analogues are not described in that paper. The former was obtained at Porton Down after two recrystallizations from ethanol as salmon pink needles (mp 141°C) and the latter similarly as cream crystals (mp 90–91°C). The four BMNs examined in the historic trial were analytically pure.

CS was chosen as most closely meeting the requirements for a riot control agent by Porton scientists in 1958 by exposing human volunteers to known concentrations of the most promising candidates in a 100 m^3^ chamber [4]. This time the agent dissolved in ethanol (15 ml) was sprayed to produce a concentration of 0.1–1.0 ppm in the chamber. Subjects were introduced into the chamber after a preliminary rest, and the nature, timing and severity of their reactions observed under conditions of moderate exercise. The volunteers, members of the UK Armed Forces, ranged in age from 18 to 30; for comparative trials, they were randomly selected in groups of 6 or 7, and their activity during exposure comprised walking briskly round the chamber. They wore standard denim working dress. The importance and purpose of the tests were explained to them before exposure and it was considered that ‘their standard of discipline and self-respect, especially in the presence of their fellows, provided a degree of psychological resistance and determination which was probably comparable to that of a well-motivated rioter’. Subsequent exposures were conducted at higher concentrations, such as 1 ppm, depending on the degree of physiological response. Each group of volunteers was exposed to several different agents or to several different concentrations of the same agent. The information obtained was used to classify the compounds into three categories according to the nature and severity of their physiological effects: not affected, harassed or incapacitated. Harassed was the concentration that compelled subjects to wear respirators, and incapacitated the state where they were unable to perform any task.

At 0.1–1.0 ppm, the most outstanding compounds in terms of speed of action and severity of effect were the 2-Cl, 2-NO_2_, 2-F and 2-Br analogues. The 2-F analogue was less potent than CS; it had a greater effect on the throat, but its lachrymatory effect was much lower and it caused no pain in the eyes. Other active compounds were the 2,6-di-Cl, 3-OH, 3-Br, 3-CN and 2-CN analogues. The 2-Cl-3-OH, 2,4-di-Cl, BMN, 1-naphthyl, 3-NO_2_, 4-NO_2_, 4-Cl, 2-OEt, 3,4-di-OEt and 4-CN analogues had a weak irritant action insufficient to harass the subjects. It is difficult to explain why CS caused lachrymation but its 2-F analogue did not. As both were described as being generated as aerosols (but not analysed as such), it is possible that a trace of CS entered the eye but the 2-F analogue did not; direct application of both compounds to the eye was not performed, but would have allowed a better comparison of the lachrymatory response. The irritancy of a compound to the cornea, nasal mucosa and skin might depend on the pH of these organs. The pH of the ocular surface [49], nasal cavity [50] and epidermis [51] in humans is 7.1, 6.3 and 6.0–4.0, respectively.

**Figure 3.**
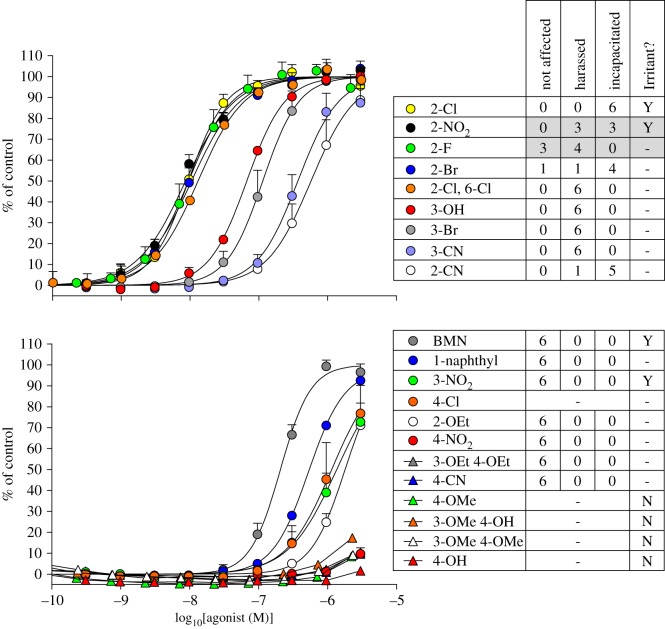
hTRPA1 agonist potency correlates with irritancy to humans. Left panels show concentration–response relationships for the indicated analogues of BMN. Right panels show results from human volunteer chamber trials with the analogue dispersed at 1 ppm or 0.1 ppm (shaded) and exposure lasting approximately 1–3 min. Results show numbers of volunteers (six or seven) in the indicated categories [4]. Human irritancy of BMN and its analogues described in the literature [1,2] is also shown (Irritant?). Blanks indicate the compound was not tested. The 4-Cl analogue is included as it was synthesized by Crichton *et al*. [4] but did not proceed to chamber trials due to a lack of irritancy in initial tests. The discrepancy between the powerful physiological effect of the 2-CN analogue and its low hTRPA1 agonist activity—the only anomaly in our correlation—may arise from a rapid rate of hydrolysis, which diminished its concentration during the cell-based fluorescence assay.

Despite inter-volunteer variability, the physiological potency of the BMNs determined in the trials correlates well with their EC_50_ values from the hTRPA1 assay (figure 3). This highlights the importance of the hTRPA1 channel as an early warning system for potentially harmful chemicals and reveals that lachrymation, sternutation and skin irritation are all defensive responses triggered by its activation. Activation of TRPA1 evidently unifies the separate physiological reactions to chemical irritants. The correlation suggests a high selectivity for the BMNs for this channel (CS does not activate hTRPV1 or cTRPM8 ion channels [13]).

The presence of hTRPA1 mRNA in human trigeminal ganglia supports the presence of this receptor target for CS in the sensory nerve endings of the eye [13]. The sneezing, cough, mucus secretion and bronchoconstriction experienced by the volunteers exposed to aerosols of the most potent BMNs are components of a coordinated response to expel the irritants from the respiratory system. Activation of the hTRPA1 receptors in the sensory nerves localized from the nose to the lower airways below the epithelium presumably initiate the protective reflex responses, including cough [52]. Human volunteer trials involving aerosols of *trans*-cinnamaldehyde have shown that inhalation of this hTRPA1 agonist, like the potent BMNs, provokes coughing [28,29]. Respiratory depression, as a result of subjects exposed to aerosolized irritants holding their breath, is also a defensive response that may originate from hTRPA1 activation [53]. The stinging experienced from the most potent BMNs landing on the skin of the volunteers during the historic Porton trials suggests activation of sensory nerve endings, which are known to be present in human skin [54].

### 4.3 Reactions of benzylidenemalononitriles with a model thiol

**Figure 4.**
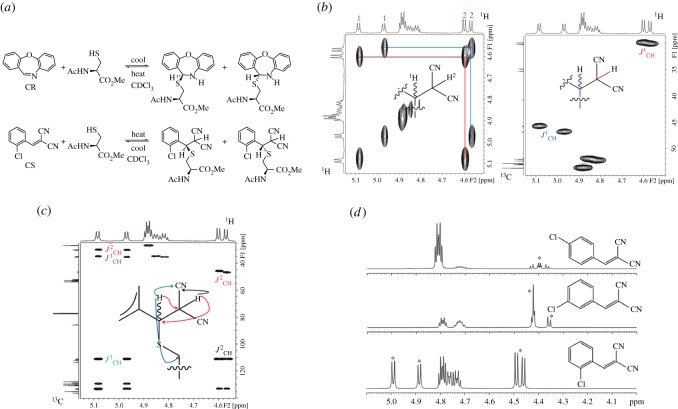
CR and CS react reversibly with *N*-acetyl-l-cysteine methyl ester in CDCl_3_. (*a*) Equilibria and product diastereomers. (*b*) ^1^H COSY (left) and ^1^H-^13^C HSQC with DEPT-135 editing (right) NMR spectra: the CH_2_ group of the diastereomers gives a negative signal. (*c*) ^1^H-^13^C HMBC NMR spectrum showing signals for the two diastereomers. (*d*) ^1^H NMR spectra of mixtures of 2- (CS), 3- and 4-Cl analogues with the cysteine ester. The substituent has a strong electronic effect on the coupling pattern of the protons of the SCHCH(CN)_2_ group of the product (marked with asterisks). A Cl atom in the 2-position produces symmetrical doublets for both diastereomers. A Cl atom in the 3-position causes these signals to overlap, but gives an integral of 3:1 consistent with the proposed structures. A Cl atom in the 4-position produces four doublets, but due to their similarity, roofing effects are observed. These types of coupling patterns were seen across all the substituents and positions studied.

It is not known whether the reactive cysteine of hTRPA1 combines with BMNs in a hydrophobic or hydrophilic environment. To understand how it interacts in the former, we monitored the reaction of a model thiol with CR, CS and a selection of BMNs of different agonist potencies. Equimolar amounts of *N*-acetyl-l-cysteine methyl ester and BMNs were combined in deuterated chloroform (CDCl_3_) and ^1^H NMR measurements made sequentially. Spectra recorded immediately after mixing at 25°C showed CR and CS to react the quickest and most completely (37% and 23%, respectively). The starting materials and products attained equilibrium rapidly. Reversibility was noted upon heating from 25°C to 37°C and then cooling to 25°C (electronic supplementary material, table S1). At physiological temperature the position of equilibrium between CR, thiol and product shifted towards the reactants, and returned towards the product upon cooling to 25°C (figure 4*a*). The singlet at *δ* 8.56 ppm for the CH=N proton of CR decreased as a doublet appeared at *δ* 5.45 ppm for the SCHN group of the product. The singlet at *δ* 8.30 ppm for the CH=C(CN)_2_ proton of CS decreased similarly and was replaced by a doublet of doublets at *δ* 5.08 and one at *δ* 4.95 ppm. These data agree with those from reaction of benzylthiol with CR and CS in deuterated DMSO [13], but with one important difference: the doublet of doublets seen corresponded to two diastereomers, rather than the single product observed with benzylthiol, due to the presence of two chiral centres in the product. Exact assignment of signals to each diastereomer could not be achieved at this stage (NOE and ROSEY NMR experiments at 400 MHz failed to reveal significant differences between the diastereomer resonances). The isomers were confirmed by two-dimensional COSY, HSQC and HMBC methods (figure 4*b*,*c*) and proton coupling in the SCHCH(CN)_2_ groups of these isomers differed according to the position of the ring substituent (figure 4D).

**Figure 5.**
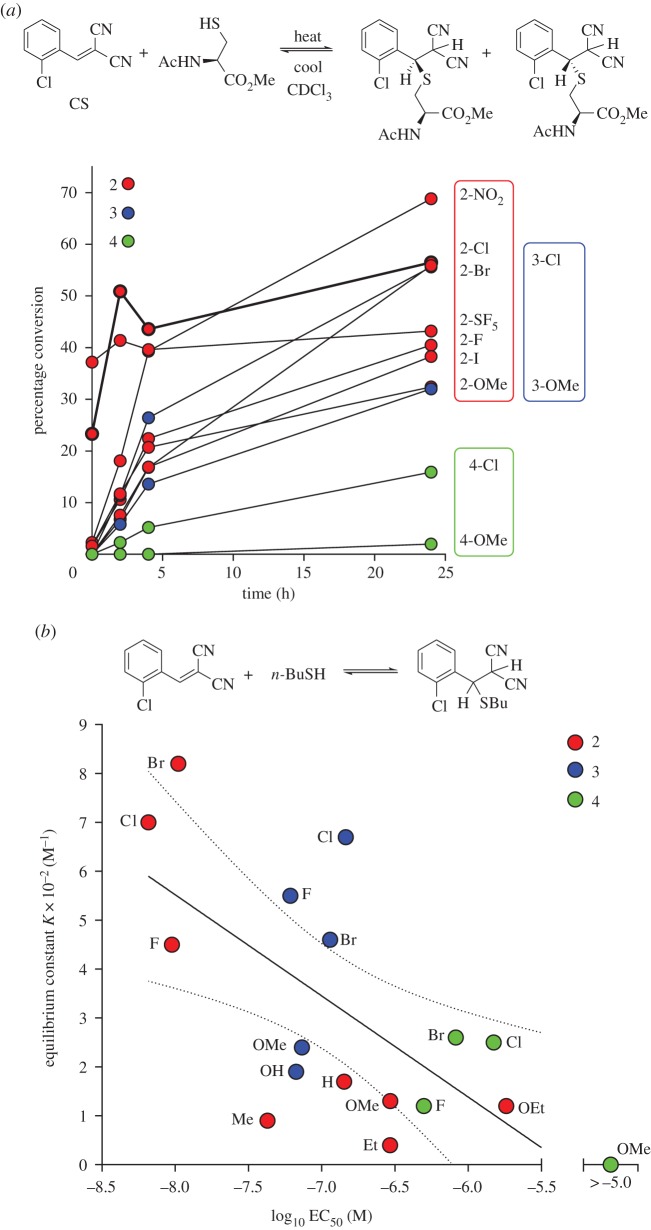
Reactivity of BMNs to thiols. Red, blue and green dots and boxes signify, respectively, 2-, 3- and 4-substituted BMNs. (*a*) BMNs add reversibly to *N*-acetyl-l-cysteine methyl ester in CDCl_3_ to produce two diastereomeric products. Percentage conversions were measured by ^1^H NMR spectroscopy: the 4 and 24 h time points were measured at 37°C and the others at 25°C (electronic supplementary material, table S1). (*b*) Equilibrium constant *K* ([BMN-thiol adduct]/[BMN] × [*n*-BuSH]) [55] for the instantaneous reaction of BMNs with *n*-butanethiol at 25°C in 20% ethanol–80% phosphate pH 7 buffer (v/v) compared with our log EC_50_ data for hTRPA1 activation by the BMNs at 25°C (electronic supplementary material, table S2). The highest equilibrium constants were associated generally with the more potent TRPA1 agonists.

With CS and analogues, the position of equilibrium shifted towards product on warming (the 2-SF_5_ analogue behaved anomalously, and as with CR, the reaction reversed on heating). Spectra recorded immediately after mixing indicated the reactivity order: CR (39%) > 2-SF_5_ (37%) > CS (23%) > 2-NO_2_ (2% conversion to product). The other analogues reacted more slowly. The time profiles in figure 5*a* indicate that the reactivity towards the thiol declined in the order 2- > 3- > 4-substituted analogues, reflecting the hTRPA1 agonist potencies (figure 5*a*).

### 4.4 Mechanism of hTRPA1 activation

To understand how the constitution of the BMNs affected hTRPA1 activation, we plotted the equilibrium constants [55] of some BMNs with *n*-butanethiol in aqueous solution against our EC_50_ values (figure 5*b*). This assumed that the agonist response was rapid and reversible. The reported equilibrium constants for the 2- and 3-halogen analogues exceeded those of the corresponding 4-analogues, and for the methoxy-BMNs declined in the order 3-OMe > 2-OMe > 4-OMe, paralleling the agonist potencies (table 1). However, the thiol equilibria span an order of magnitude, whereas the EC_50_ values span three orders of magnitude, suggesting that factors other than the thiol interaction are important. The BMN must enter the receptor and approach the thiol closely, and steric influences governing access may be considerable, in line with the hypothesis for CR analogues that a lack of steric hindrance is a key driver for potency [15].

With these considerations, the structure–irritancy relationships of the BMNs and their mechanism can be rationalized. The requirement for aqueous solubilization of the most potent BMNs to cause irritation is supported by the fact that dry CS does not irritate human skin [10]. Stinging of the skin by CS is only felt in the presence of moisture and intensifies upon perspiration, lachrymation or rhinorrhoea [5,8,10]. Symptoms can occur repeatedly after skin exposure upon wetting [56] for up to 24 h, although with decreasing severity over time. The solubility of CS in water is approximately 4 mg per 100 ml [57,58], a concentration that greatly exceeds its EC_50_ for hTRPA1 activation. The influence of the equilibrium constant of the reaction between the BMNs and receptor on the gating of the channel is not understood. There must be a way of deactivating the channel as the irritation caused by physiologically active BMNs fades within 10 min once humans leave a contaminated area and no permanent harm is evident [8]. Hydrolysis of BMNs [59] or their reaction with cellular thiols such as glutathione, which can yield stable adducts [60], may enable hTRPA1 to reset. Reaction of the hTRPA1 cysteine residue with irritant BMNs might help explain why pain is felt immediately, continues during exposure with an intensity proportional to their concentration, disappears within a short time following termination of exposure, recommences on renewed exposure and after sufficiently long or repeated exposure fades or is tolerated [8] (which suggests desensitization). The activation of the hTRPA1 receptors in the various affected tissues is then presumed to cause the different physiological responses and pain perceptions by the pathways summarized in the Introduction.

## 5. Conclusion

We have provided evidence that TRPA1 is a receptor for irritants on nociceptive neurons involved in pain perception in humans [61] and probably related species [62]. It is revealed as an early warning system against potentially harmful chemicals entering the body and the trigger for the defensive reflexes of lachrymation, sneezing, coughing and skin irritation. Aqueous dissolution and thiolation, size-permitting, explains how solid BMNs, and probably other solid chemical irritants, discomfort humans. These insights should help research to counter diseases involving hTRPA1-mediated pain and those including symptoms of lachrymation, sneezing, coughing and skin irritation. A generic approach to the treatment of such diseases is indicated by the correlation between channel activation and physiological response presented herein. We have shown that the irritancy for a subset of BMNs—those whose human irritancy has been assessed—correlates with hTRPA1 antagonism. The BMNs synthesized, spanning a range of agonist potencies, provide a pharmacological toolset that could be used to delineate differences of function across TRPA1 ion channels of different animal species. This would help solve the long-standing problem of why some animal species are not irritated by CS in the same way as humans and help address the serious challenge of identifying suitable surrogate species for humans in preclinical trials of hTRPA1 antagonists.

## Supplementary Material

Synthetic data Supplementary figure Supplementary table 1 Supplementary table 2

## Data accessibility

The datasets supporting this article have been uploaded as electronic supplementary material.
