# Supplementary material for: Potency of irritation by benzylidenemalononitriles in humans correlates with TRPA1 ion channel activation
Source: R Soc Open Sci. 2015 Jan 28;2(1):140160. doi: 10.1098/rsos.140160 (PMC4448789; doi:10.1098/rsos.140160)
Supplement: Synthetic data Supplementary figure Supplementary table 1 Supplementary table 2 [file rsos140160supp1.docx]

**Supplementary Information**

*Benzylidenemalononitrile (BMN).* Method A. Pale yellow solid (93%). Mp 83 °C (lit. 84 °C [2]). ¹H NMR (400 MHz, CDCl_3_) 7.93-7.90 (2H, m), 7.79 (1H, s), 7.67-7.61 (1H, m), 7.57-7.52 (2H, m). ^13^C{^1^H} NMR (100 MHz, CDCl_3_) 160, 134, 131, 130, 129, 113, 112, 83. LRI 1459, *m/z* (%): 154 (100), 127 (81), 103 (77), 100 (17), 76 (22), 75 (16), 74 (11), 63 (11), 51 (14), 50 (14).

*2-Fluorobenzylidenemalononitrile (2-F).* Method A. Pale cream solid (93%). Mp 120 °C (lit. 119 °C [38]). ¹H NMR (400 MHz, CDCl_3_) 8.31-8.26 (1H, m), 8.11 (1H, s), 7.68-7.62 (1H, m), 7.34 (1H, dd, *J* = 7.7, 7.7 Hz), 7.27-7.21 (1H, m). ^13^C{^1^H} NMR (100 MHz, CDCl_3_) 162, 160, 151, 136, 128, 125, 119, 116, 113, 112, 84. ^19^F{^1^H} NMR (376 MHz, CDCl_3_) -111. LRI 1391, *m/z* (%): 173 (11), 172 (100), 146 (13), 145 (99), 121 (48), 100 (11), 99 (14), 94 (19), 75 (20), 74 (13).

*3-Fluorobenzylidenemalononitrile (3-F).* Method A. Pale cream solid (98%). Mp 93 °C. ¹H NMR (400 MHz, CDCl_3_) 7.78 (1H, s), 7.72-7.64 (2H, m), 7.60-7.53 (1H, m), 7.40-7.34 (1H, m). ^13^C{^1^H} NMR (100 MHz, CDCl_3_) 164, 161, 158, 132, 131, 126, 121.7, 121.5, 117, 116, 113, 112, 84. ^19^F{^1^H} NMR (376 MHz, CDCl_3_) -109. LRI 1433, *m/z* (%): 173 (12), 172 (100), 145 (81), 121 (71), 100 (13), 99 (13), 94 (23), 75 (28), 74 (14), 50 (13).

*4-Fluorobenzylidenemalononitrile (4-F).* Method A. Pale cream solid (96%). Mp 129 °C. ¹H NMR (400 MHz, CDCl_3_) 7.99-7.94 (2H, m), 7.74 (1H, s), 7.27-7.22 (2H, m). ^13^C{^1^H} NMR (100 MHz, CDCl_3_) 167, 164, 158, 133, 127, 117, 113, 112, 82. ^19^F{^1^H} NMR (376 MHz, CDCl_3_) -100. LRI 1451, *m/z* (%): 172 (100), 145 (77), 121 (75), 99 (13), 94 (21), 75 (27), 74 (14), 62 (12), 57 (12), 50 (12).

*2-Chlorobenzylidenemalononitrile (2-Cl or CS).* Method A. Cream solid (90%). Mp 95 °C (lit. 95 °C [2]). ¹H NMR (600 MHz, CDCl_3_) 8.30 (1H, s, *α*-H), 8.21 (1H, d, *J* = 7.7 Hz), 7.59-7.58 (2H, m), 7.51-7.45 (1H, m). ^13^C{^1^H} NMR (150 MHz, CDCl_3_) 156 (*α*-C), 136, 135, 130, 129.5, 129, 127, 113, 112, 86. LRI 1554, *m/z* (%): 188 (41), 161 (15), 153 (100), 126 (21), 100 (19), 99 (24), 76 (15), 75 (30), 74 (18), 50 (13).

*3-Chlorobenzylidenemalononitrile (3-Cl).* Method A. Recrystallised from hot ethanol to give pale cream needles (93%). Mp 116 °C (lit. 116 °C [39]). ¹H NMR (400 MHz, CDCl_3_) 7.85-7.83 (2H, m), 7.73 (1H, s), 7.62-7.60 (1H, m), 7.50 (1H, dd, *J* = 8.2, 8.2 Hz). ^13^C{^1^H} NMR (100 MHz, CDCl_3_) 158, 135, 134, 132, 130.9, 130.4, 128, 113, 112, 85. LRI 1626, *m/z* (%): 190 (19), 188 (54), 161 (23), 153 (100), 137 (25), 126 (22), 100 (21), 99 (20), 75 (31), 74 (20).

*4-Chlorobenzylidenemalononitrile (4-Cl).* Method A. Recrystallised twice from hot ethanol to give white crystals (58%). Mp 162 °C (lit. 163 °C [38]). ¹H NMR (400 MHz, CDCl_3_) 7.86 (2H, dd, *J* = 1.7, 6.8 Hz), 7.73 (1H, s), 7.53 (2H, dd, *J* = 1.9, 6.7 Hz). ^13^C{^1^H} NMR (100 MHz, CDCl_3_) 158, 141, 131, 130, 129, 113, 112, 83. LRI 1647, *m/z* (%): 190 (23), 188 (72), 161 (30), 153 (100), 137 (30), 126 (26), 100 (23), 99 (26), 75 (40), 74 (24).

*2-Bromobenzylidenemalononitrile (2-Br).* Method A. Recrystallised from hot ethanol to give pale cream crystals (86%). Mp 91 °C (lit. 90 °C [39]). ¹H NMR (400 MHz, CDCl_3_) 8.22 (1H, s), 8.13 (1H, dd, *J* = 1.8, 7.6 Hz), 7.75 (1H, dd, *J* = 1.5, 7.8 Hz), 7.52-7.43 (2H, m). ^13^C{^1^H} NMR (100 MHz, CDCl_3_) 158, 135, 134, 130, 129, 128, 126, 113, 112, 86. LRI 1651, *m/z* (%): 234 (19), 232 (20), 154 (12), 153 (100), 126 (28), 100 (11), 99 (12), 76 (8), 75 (12), 50 (9).

*3-Bromobenzylidenemalononitrile (3-Br).* Method A. Rinsed with isopropanol then petroleum ether (bp 40-60 °C) to give a cream solid (64%). Mp 110 °C (lit. 110 °C [39]). ¹H NMR (600 MHz, CDCl_3_) 7.97 (1H, s), 7.91 (1H, d, *J* = 7.9 Hz), 7.76 (1H, d, *J* = 7.5 Hz), 7.71 (1H, s), 7.44 (1H, dd, *J* = 8.0, 8.0 Hz). ^13^C{^1^H} NMR (150 MHz, CDCl_3_) 158, 137, 133, 132, 131, 128, 123, 113, 112, 84. LRI 1730, *m/z* (%): 234 (31), 232 (31), 153 (100), 126 (37), 100 (18), 99 (17), 76 (16), 75 (19), 51 (12), 50 (16).

*4-Bromobenzylidenemalononitrile (4-Br).* Method B. Rinsed with isopropanol then petroleum ether (bp 40-60 °C) to give a cream solid (73%). Mp 164 °C. ¹H NMR (400 MHz, CDCl_3_) 7.80 (2H, m), 7.75-7.70 (3H, m). ^13^C{^1^H} NMR (100 MHz, CDCl_3_) 158, 133, 131, 129.9, 129.6, 113, 112, 83. LRI 1753, *m/z* (%): 234 (41), 232 (38), 153 (100), 126 (42), 100 (19), 99 (18), 76 (18), 75 (23), 74 (14), 50 (20).

*2-Iodobenzylidenemalononitrile (2-I).* Method B. Recrystallised from hot ethanol to give a sand-coloured powder (60%). Mp 100 °C. ¹H NMR (400 MHz, CDCl_3_) 8.09 (1H, s), 8.07-8.03 (2H, m), 7.56 (1H, dd, *J* = 7.7, 7.7 Hz), 7.32-7.27 (1H, m). ^13^C{^1^H} NMR (100 MHz, CDCl_3_) 163, 140, 134.6, 134, 129.7, 129, 113, 112, 101, 86. LRI 1773, *m/z* (%): 280 (36), 154 (10), 153 (100), 127 (13), 126 (37), 100 (13), 99 (14), 75 (13), 51 (10), 50 (10).

*3-Iodobenzylidenemalononitrile (3-I).* Method B. Rinsed with isopropanol then petroleum ether (bp 40-60 °C) to give a pale orange solid (58%). Mp 103-104 °C (lit. 107-108 °C [39]). ¹H NMR (400 MHz, CDCl_3_) 8.15 (1H, t, *J* = 1.7 Hz), 8.00-7.95 (2H, m), 7.70 (1H, s), 7.31 (1H, dd, *J* = 7.9, 7.9 Hz). ^13^C{^1^H} NMR (100 MHz, CDCl_3_) 158, 143, 139, 132, 131, 128.9, 113, 112, 94.9, 84. LRI 1862, *m/z* (%): 280 (70), 154 (12), 153 (100), 127 (29), 126 (50), 100 (19), 99 (16), 76 (13), 75 (17), 50 (15).

*4-Iodobenzylidenemalononitrile (4-I).* Method B. Rinsed with isopropanol then petroleum ether (bp 40-60 °C) to give a cream solid (68%). Mp 150-151 °C. ¹H NMR (400 MHz, CDCl_3_) 7.93 (2H, d, *J* = 8.5 Hz), 7.72 (1H, s), 7.63 (2H, d, *J* = 8.3 Hz). ^13^C{^1^H} NMR (100 MHz, CDCl_3_) 158, 139, 131, 130, 113, 112, 102, 83. LRI 1891, *m/z* (%): 280 (65), 153 (100), 127 (46), 126 (75), 100 (26), 99 (24), 76 (19), 75 (25), 51 (22), 50 (28).

*2-Nitrobenzylidenemalononitrile (2-NO_2_).* Method A. Recrystallised twice from hot ethanol to give coffee-coloured needles (94%). Mp 140 °C (lit. 138 °C [38]). ¹H NMR (400 MHz, CDCl_3_) 8.45 (1H, s), 8.38-8.35 (1H, m), 7.91-7.86 (1H, m), 7.84-7.79 (2H, m). ^13^C{^1^H} NMR (100 MHz, CDCl_3_) 158, 146, 134, 133, 130, 126, 125, 112, 111, 88. LRI 1747, *m/z* (%): 126 (55), 119 (34), 114 (54), 100 (35), 99 (32), 92 (100), 91 (33), 76 (32), 75 (34), 51 (31).

*3-Nitrobenzylidenemalononitrile (3-NO_3_).* Method A. Recrystallised thrice from hot ethanol to give dark cream crystals (46%). Mp 105 °C (lit. 105 °C [2]). ¹H NMR (600 MHz, CDCl_3_) 8.67 (1H, s), 8.48 (1H, d, *J* = 8.1 Hz), 8.33 (1H, d, *J* = 7.7 Hz), 7.92 (1H, s), 7.80 (1H, dd, *J* = 8.0, 8.0 Hz). ^13^C{^1^H} NMR (150 MHz, CDCl_3_) 157, 148, 134.9, 134.7, 132, 131, 128, 125, 112, 111, 86. LRI 1892, *m/z* (%): 199 (61), 153 (100), 141 (30), 126 (82), 114 (21), 100 (32), 99 (28), 75 (30), 51 (24), 50 (26).

*4-Nitrobenzylidenemalononitrile (4-NO_2_).* Method A. Recrystallised from hot ethanol to give a sand-coloured powder (88%). Mp 161 °C (lit. 160 °C [38]). ¹H NMR (400 MHz, CD_3_CN) 8.38 (2H, d, *J* = 8.9 Hz), 8.25 (1H, s), 8.11 (2H, d, *J* = 8.9 Hz). ^13^C{^1^H} NMR (100 MHz, CD_3_CN) 159, 150, 137, 131, 125, 113, 112, 87. LRI 1888, *m/z* (%): 199 (65), 153 (66), 141 (79), 126 (100), 114 (49), 100 (41), 99 (37), 75 (42), 51 (33), 50 (36).

*2-Cyanobenzylidenemalononitrile (2-CN).* 2-Cyanobenzaldehyde (5.37 g, 41 mmol) was added to a stirred mixture of malononitrile (2.60 g, 39 mmol) and absolute ethanol (20 ml). The precipitate that formed was filtered off, washed with cold ethanol, and dried under vacuum to give a cream solid (5.80 g, 82%). Mp 127 °C (lit. 130 °C [4]). ¹H NMR (400 MHz, CDCl_3_) 8.43 (1H, d, *J* = 8.1 Hz), 8.21 (1H, s), 7.92-7.83 (2H, m), 7.80-7.77 (1H, m). ^13^C{^1^H} NMR (100 MHz, CDCl_3_) 154, 134.1, 134, 133.7, 132.7, 128, 115, 114, 112, 111, 88. LRI 1655, *m/z* (%): 179 (75), 152 (100), 128 (16), 125 (14), 100 (16), 99 (16), 76 (20), 75 (27), 51.1 (15), 50 (20).

*3-Cyanobenzylidenemalononitrile (3-CN).* Method B. Rinsed with isopropanol then petroleum ether (bp 40-60 °C) to give an off-white solid (85%). Mp 149 °C (lit. 149 °C [4]). ¹H NMR (600 MHz, CDCl_3_) 8.21 (1H, d, *J* = 8.1 Hz), 8.08 (1H, s), 7.91 (1H, d, *J* = 7.9 Hz), 7.80 (1H, s), 7.72 (1H, dd, *J* = 8.0, 8.0 Hz). ^13^C{^1^H} NMR (150 MHz, CDCl_3_) 157, 137.2, 137.0, 136.9, 133.9, 133.5, 131, 130, 116.9, 114, 112.7, 111.6, 86. LRI 1786, *m/z* (%): 180 (13), 179 (100), 153 (14), 152 (92), 128 (37), 125 (12), 101 (15), 76 (17), 75 (22), 50 (14).

*4-Cyanobenzylidenemalononitrile (4-CN).* Method B. Rinsed with isopropanol then petroleum ether (bp 40-60 °C) to give an off-white solid (95%). Mp 154 °C (lit. 154 °C [4]). ¹H NMR (600 MHz, CDCl_3_) 8.00 (2H, d, *J* = 8.5 Hz), 7.85-7.83 (3H, m). ^13^C{^1^H} NMR (150 MHz, CDCl_3_) 157, 134, 133, 130, 117, 112, 111, 87. LRI 1778, *m/z* (%): 180 (13), 179 (100), 153 (13), 152 (92), 128 (38), 125 (12), 101 (15), 76 (17), 75 (21), 50 (15).

*2-(Trifluoromethyl)benzylidenemalononitrile (2-CF_3_).* Method A. Cream needles (92%). Mp 47 °C. ¹H NMR (400 MHz, CDCl_3_) 8.24-8.22 (1H, m), 8.08 (1H, d, *J* = 6.9 Hz), 7.87-7.83 (1H, m), 7.77-7.72 (2H, m). ^13^C{^1^H} NMR (100 MHz, CDCl_3_) 156, 133, 132, 130, 129.7, 129.4, 129.1, 128.6, 127, 126, 124, 121, 119, 112, 111, 88. ^19^F{^1^H} NMR (376 MHz, CDCl_3_) -58. LRI 1317, *m/z* (%): 168 (36), 142 (12), 141 (100), 140 (56), 114 (34), 113 (13), 89 (12), 63 (18), 51 (15), 50 (12).

*3-(Trifluoromethyl)benzylidenemalononitrile (3-CF_3_).* Method A. Recrystallised from hot ethanol to give pale cream crystals (60%). Mp 83 °C. ¹H NMR (400 MHz, CDCl_3_) 8.20 (1H, d, *J* = 7.9 Hz), 8.05 (1H, s), 7.89 (1H, d, *J* = 7.9 Hz), 7.84 (1H, s), 7.72 (1H, dd, *J* = 7.9, 7.9 Hz). ^13^C{^1^H} NMR (100 MHz, CDCl_3_) 158, 132.7, 132.5, 132.1, 131.3, 130.7, 130.4, 127.7, 124, 121, 113, 112, 85. ^19^F{^1^H} NMR (376 MHz, CDCl_3_) -63. LRI 1429, *m/z* (%): 222 (100), 203 (27), 195 (37), 172 (22), 171 (26), 153 (29), 126 (24), 99 (25), 75 (33), 69 (20).

*4-(Trifluoromethyl)benzylidenemalononitrile (4-CF_3_).* Method A. White solid (96%). Mp 113 °C. ¹H NMR (600 MHz, CDCl_3_) 8.02 (2H, d, *J* = 8.3 Hz), 7.85 (1H, s), 7.81 (2H, d, *J* = 8.5 Hz). ^13^C{^1^H} NMR (150 MHz, CDCl_3_) 158, 135, 133, 130, 126, 125, 124, 122, 120, 113, 111, 86. ^19^F{^1^H} NMR (376 MHz, CDCl_3_) -63. LRI 1426, *m/z* (%): 222 (100), 203 (31), 195 (54), 171 (32), 153 (43), 145 (23), 126 (24), 100 (21), 99 (24), 75 (34).

*3-Hydroxybenzylidenemalononitrile (3-OH).* Method A. Recrystallised twice from hot ethanol to give yellow crystals (28%). Mp 152 °C (lit. 153 °C [4]). ¹H NMR (400 MHz, CD_3_CN) 8.04 (1H, s), 7.56 (1H, s), 7.47-7.38 (3H, m), 7.15-7.12 (1H, m). ^13^C{^1^H} NMR (100 MHz, CD_3_CN) 161, 158, 133, 131, 123, 122, 116, 114, 113, 83. LRI 1468, *m/z* (%): 298 (64), 190 (88), 171 (43), 163 (100), 151 (27), 144 (29), 139 (45), 124 (37), 127 (34), 89 (47).

*4-Hydroxybenzylidenemalononitrile (4-OH).* Method A. Crude product dissolved in acetonitrile and refrigerated to produce lemon crystals (88%). Mp 190 °C (lit. 190 °C [2]). ¹H NMR (400 MHz, CD_3_CN) 7.95 (1H, s), 7.93-7.88 (2H, m), 7.03-7.00 (2H, m). ^13^C{^1^H} NMR (100 MHz, CD_3_CN) 163, 160, 134, 124, 117, 115, 114, 77. LRI 1916, *m/z* (%): 170 (100), 143 (22), 142 (33), 119 (45), 115 (37), 114 (29), 91 (27), 88 (20), 63 (19), 62 (15).

*2-(Difluoromethoxy)benzylidenemalononitrile (2-OCF_2_H).* Method A. Fine creamy-orange powder (98%). Mp 55 °C. ¹H NMR (400 MHz, CDCl_3_) 8.28 (2H, dd, *J* = 1.4, 8.0 Hz), 8.19 (1H, s), 7.68-7.62 (1H, m), 7.42-7.37 (1H, m), 7.30-7.26 (2H, m), 6.64 (1H, t, *J* = 72.4 Hz). ^13^C{^1^H} NMR (100 MHz, CDCl_3_) 153, 150, 135, 129, 126, 123, 119, 118, 115, 113, 112.6, 112.2, 85. ^19^F{^1^H} NMR (376 MHz, CDCl_3_) -81. LRI 1521, *m/z* (%): 220 (43), 219 (17), 170 (50), 143 (100), 115 (36), 114 (39), 88 (22), 63 (18), 51 (47), 50 (13).

*2-Methoxybenzylidenemalononitrile (2-OMe).* Method A. Sunflower-yellow solid (91%). Mp 84 °C (lit. 84 °C [2]). ¹H NMR (400 MHz, CDCl_3_) 8.31 (1H, s), 8.19 (1H, dd, *J* = 1.5, 8.0 Hz), 7.61-7.56 (1H, m), 7.08 (1H, dd, *J* = 7.7, 7.7 Hz), 6.99 (1H, d, *J* = 8.5 Hz), 3.93 (3H, s). ^13^C{^1^H} NMR (100 MHz, CDCl_3_) 159, 154, 136, 128, 121, 120, 114, 113, 111, 81, 55. LRI 1701, *m/z* (%): 184 (74), 156 (42), 141 (24), 127 (25), 119 (86), 114 (100), 91 (61), 88 (30), 78 (34), 63 (28).

*3-Methoxybenzylidenemalononitrile (3-OMe).* Method A. Pale cream crystals (84%). Mp 106 °C (lit. 104 °C [38]). ¹H NMR (400 MHz, CDCl_3_) 7.75 (1H, s), 7.49 (1H, s), 7.47-7.43 (2H, m), 7.19-7.16 (1H, m), 3.87 (3H, s). ^13^C{^1^H} NMR (100 MHz, CDCl_3_) 160, 159, 132, 130, 124, 121, 114, 113, 112, 83, 55. LRI 1665, *m/z* (%): 184 (100), 156 (35), 155 (33), 154 (20), 141 (32), 127 (56), 114 (74), 103 (22), 88 (20), 63 (23).

*4-Methoxybenzylidenemalononitrile (4-OMe).* Method A. Recrystallised twice from hot ethanol to give pale yellow needles (63%). Mp 114 °C (lit. 114 °C [2]). ¹H NMR (400 MHz, CDCl_3_) 7.92 (2H, dd, *J* = 1.9, 7.0 Hz), 7.66 (1H, s), 7.02 (2H, dd, *J* = 2.0, 7.0 Hz), 3.92 (3H, s). ^13^C{^1^H} NMR (100 MHz, CDCl_3_) 164, 158, 133, 124, 115, 114, 113, 78, 55. LRI 1786, *m/z* (%): 185 (12), 184 (100), 169 (12), 141 (36), 133 (15), 115 (10), 114 (65), 88 (16), 63 (16), 62 (10).

*2-Ethoxybenzylidenemalononitrile (2-OEt).* Method B. Rinsed with isopropanol then petroleum ether (bp 40-60 °C) to give a pale yellow solid (70%). Mp 77-78 °C (lit. 80 °C [4]). ¹H NMR (600 MHz, CDCl_3_) 8.34 (1H, s), 8.21 (1H, dd, *J* = 1.6, 8.1 Hz), 7.56 (1H, dd, *J* = 7.6, 7.6 Hz), 7.06 (1H, dd, *J* = 7.6, 7.6 Hz), 6.96 (1H, d, *J* = 8.5 Hz), 4.14 (2H, q, *J* = 7.0 Hz), 1.49 (3H, t, *J* = 7.0 Hz). ^13^C{^1^H} NMR (150 MHz, CDCl_3_) 158, 154, 136, 128, 121, 120, 114, 113, 112, 81, 64, 14. LRI 1734, *m/z* (%): 198 (21), 183 (19), 170 (26), 144 (10), 143 (100), 115 (25), 114 (22), 88 (11), 63 (8), 51 (7).

*2,3-Dichlorobenzylidenemalononitrile (2,3-di-Cl).* Method B. Rinsed with isopropanol then petroleum ether (bp 40-60 °C) to give a fine white powder (86%). Mp 104-105 °C. ¹H NMR (600 MHz, CDCl_3_) 8.28 (1H, s), 8.04 (1H, d, *J* = 7.9 Hz), 7.73 (1H, d, *J* = 8.1 Hz), 7.44 (1H, dd, *J* = 8.1, 8.1 Hz). ^13^C{^1^H} NMR (150 MHz, CDCl_3_) 156, 135, 134.9, 134, 131, 128, 127, 112, 111, 87. LRI 1725, *m/z* (%): 224 (29), 222 (45), 189 (33), 187 (100), 152 (18), 151 (17), 124 (25), 99 (19), 75 (21), 74 (17).

*2,4-Dichlorobenzylidenemalononitrile (2,4-di-Cl).* Method B. Rinsed with isopropanol then petroleum ether (bp 40-60 °C) to give a fine cream solid (87%). Mp 149-150 °C (lit. 148-150 °C [4]). ¹H NMR (600 MHz, CDCl_3_) 8.21 (1H, s), 8.18 (1H, d, *J* = 8.7 Hz), 7.61 (1H, s), 7.47 (1H, d, *J* = 8.5 Hz). ^13^C{^1^H} NMR (150 MHz, CDCl_3_) 154, 141, 137, 130.7, 130.1, 128, 127, 113, 111, 86. LRI 1701, *m/z* (%): 224 (44), 222 (69), 189 (31), 187 (100), 152 (20), 124 (31), 100 (22), 99 (23), 75 (26), 74 (22).

*2,5-Dichlorobenzylidenemalononitrile (2,5-di-Cl).* Method B. Rinsed with isopropanol then petroleum ether (bp 40-60 °C) to give a greenish-brown solid (68%). Mp 87-88 °C. ¹H NMR (400 MHz, CDCl_3_) 8.19 (1H, s), 8.13 (1H, d, *J* = 2.2 Hz), 7.54-7.50 (2H, m). ^13^C{^1^H} NMR (100 MHz, CDCl_3_) 154, 134.7, 134.3, 134.0, 131, 130, 129, 112, 111, 87. LRI 1691, *m/z* (%): 224 (45), 222 (69), 189 (32), 187 (100), 152 (22), 124 (30), 100 (18), 99 (20), 75 (23), 74 (18).

*2,6-Dichlorobenzylidenemalononitrile (2,6-di-Cl).* Method A. White solid recrystallised twice from hot ethanol (93%). Mp 79 °C. ¹H NMR (400 MHz, CDCl_3_) 7.95 (1H, s), 7.48-7.39 (3H, m). ^13^C{^1^H} NMR (100 MHz, CDCl_3_) 156, 134, 133, 128, 111, 110, 94. LRI 1633, *m/z* (%): 224 (31), 222 (46), 189 (32), 187 (100), 152 (15), 151 (18), 124 (22), 100 (15), 99 (16), 75 (16).

*3,4-Dichlorobenzylidenemalononitrile (3,4-di-Cl).* Method B. Rinsed with isopropanol then petroleum ether (bp 40-60 °C) to give a cream solid (70%). Mp 151-152 °C. ¹H NMR (400 MHz, CDCl_3_) 7.96 (1H, d, *J* = 2.2 Hz), 7.84 (1H, dd, *J* = 2.1, 8.4 Hz), 7.71 (1H, s), 7.66 (1H, d, *J* = 8.5 Hz). ^13^C{^1^H} NMR (100 MHz, CDCl_3_) 157, 139, 134, 132, 131.7, 130, 129, 113, 112, 84. LRI 1820, *m/z* (%): 224 (50), 222 (79), 195 (26), 189 (32), 187 (100), 152 (25), 124 (37), 99 (26), 75 (32), 74 (27).

*3,5-Dichlorobenzylidenemalononitrile (3,5-di-Cl).* Method B. Rinsed with isopropanol then petroleum ether (bp 40-60 °C) to give a cream solid (40%). Mp 109-110 °C. ¹H NMR (400 MHz, CDCl_3_) 7.78 (2H, d, *J* = 2.0 Hz), 7.70 (1H, s), 7.63 (1H, t, *J* = 1.7 Hz). ^13^C{^1^H} NMR (100 MHz, CDCl_3_) 156, 136, 133.9, 133, 128, 112, 111, 86. LRI 1769, *m/z* (%): 224 (50), 222 (79), 195 (26), 189 (32), 187 (100), 152 (25), 124 (37), 99 (26), 75 (32), 74 (27).

*2,3,6-Trichlorobenzylidenemalononitrile (2,3,6-tri-Cl).* Method A. Recrystallised thrice from hot ethanol to give a pale cream solid (25%). Mp 84 °C. ¹H NMR (400 MHz, CDCl_3_) 7.90 (1H, s), 7.57 (1H, d, *J* = 8.8 Hz), 7.42 (1H, d, *J* = 8.8 Hz). ^13^C{^1^H} NMR (100 MHz, CDCl_3_) 156, 133.1, 133.0, 132.4, 132.1, 130, 129, 111, 110, 95. LRI 1787, *m/z* (%): 260 (19), 258 (61), 256 (64), 223 (66), 221 (100), 186 (28), 149 (16), 123 (17), 99 (21), 12 (19).

*2,6-Difluorobenzylidenemalononitrile (2,6-di-F).* Method A. Cream solid (96%). Mp 68 °C. ¹H NMR (400 MHz, CDCl_3_) 7.86 (1H, s), 7.63-7.55 (1H, m), 7.09 (2H, dd, *J* = 8.5, 8.5 Hz). ^13^C{^1^H} NMR (100 MHz, CDCl_3_) 161, 159, 148, 135, 112.8, 112.5, 112.3, 109, 91. ^19^F{^1^H} NMR (376 MHz, CDCl_3_) -104. LRI 1377, *m/z* (%): 191 (11), 190 (100), 171 (5), 164 (5), 163 (24), 139 (8), 112 (7), 99 (5), 75 (7), 63 (6).

*2,3,6-Trifluorobenzylidenemalononitrile (2,3,6-tri-F).* Method A. Pale brown solid (96%). Mp 77 °C. ¹H NMR (400 MHz, CD_3_CN) 8.12 (1H, s), 7.63-7.54 (1H, m), 7.23-7.16 (1H, m), 2.19 (1H, s). ^13^C{^1^H} NMR (100 MHz, CDCl_3_) 157, 154, 149.7, 149.2, 149.0, 147.0, 146.5, 122, 119, 113.2, 113.0, 112.8, 92. ^19^F{^1^H} NMR (376 MHz, CDCl_3_) -142, -130, -111. LRI 1347, *m/z* (%): 209 (11), 208 (100), 189 (8), 181 (24), 157 (12), 130 (7), 112 (12), 99 (8), 81 (14), 75 (9).

*2-Chloro-3-hydroxybenzylidenemalononitrile (2-Cl-3-OMe).* Method A. Recrystallised twice from hot ethanol to yield a yellow solid (16%). Mp 160 °C. ¹H NMR (400 MHz, CD_3_CN) 8.40 (1H, s), 7.94-7.93 (1H, m), 7.61 (1H, dd, *J* = 1.2, 7.8 Hz), 7.38 (1H, dd, *J* = 8.1, 8.1 Hz), 7.25 (1H, dd, *J* = 1.3, 8.2 Hz). ^13^C{^1^H} NMR (100 MHz, CD_3_CN) 157, 154, 131, 128, 121.9, 121.7, 121.3, 114, 112, 87. LRI 1748, *m/z* (%): 206 (32), 204 (100), 176 (51), 169 (39), 149 (23), 141 (91), 125 (35), 114 (69), 87 (26), 62 (22).

*3-Methoxy-4-hydroxybenzylidenemalononitrile (3-OMe-4-OH).* Method A. Lemon solid recrystallised twice from hot ethanol (23%). Mp 134 °C (lit. 134 °C [2]). ¹H NMR (400 MHz, CDCl_3_) 7.73 (1H, d, *J* = 1.9 Hz), 7.63 (1H, s), 7.31 (1H, dd, *J* =1.9, 8.3 Hz), 7.03 (1H, d, *J* = 8.3 Hz), 6.35 (1H, s), 3.99 (3H, s). ^13^C{^1^H} NMR (100 MHz, CDCl_3_) 159, 152, 129, 124, 115, 114, 113, 110, 78, 56. LRI 1868, *m/z* (%): 200 (81), 158 (10), 157 (100), 129 (25), 103 (19), 102 (64), 101 (16), 78 (17), 76 (14), 75 (20).

*3,4-Dimethoxybenzylidenemalononitrile (3,4-di-OMe).* Method A. Yellow solid (95%). Mp 147 °C. ¹H NMR (400 MHz, CDCl_3_) 7.69 (1H, d, *J* = 2.1 Hz), 7.65 (1H, s), 7.39 (1H, dd, *J* = 2.1, 8.4 Hz), 6.97 (1H, d, *J* = 8.5 Hz), 3.99 (6H, s), 3.95 (6H, s). ^13^C{^1^H} NMR (100 MHz, CDCl_3_) 159, 154, 149, 128, 124, 114, 113, 111, 110, 78, 56.3, 56.1. LRI 1946, *m/z* (%): 214 (100), 199 (19), 171 (43), 143 (27), 142 (16), 128 (22), 116 (53), 114 (24), 101 (38), 89 (15).

*3,4-Diethoxybenzylidenemalononitrile (3,4-di-OEt).* Method B. Rinsed with isopropanol then petroleum ether (bp 40-60 °C) to give fine yellow crystals (85%). Mp 103-104 °C (lit. 106 °C [4]). ¹H NMR (600 MHz, CDCl_3_) 7.67 (1H, d, *J* = 2.1 Hz), 7.62 (1H, s), 7.34 (1H, dd, *J* = 2.1, 8.5 Hz), 6.93 (1H, d, *J* = 8.5 Hz), 4.21 (2H, q, *J* = 7.1 Hz), 4.15 (2H, q, *J* = 7.0 Hz), 1.51 (3H, t, *J* = 7.0 Hz), 1.49 (3H, t, *J* = 7.1 Hz). ^13^C{^1^H} NMR (150 MHz, CDCl_3_) 159, 154, 149, 128, 124, 114, 113, 112, 111, 77.8, 64.8, 64.4, 14.5, 14.4. LRI 2028, *m/z* (%): 242 (39), 187 (12), 186 (100), 158 (36), 157 (25), 130 (32), 129 (24), 114 (17), 103 (23), 102 (24).

*2-Fluoro-4-(pentafluorosulfanyl)benzylidenemalononitrile (2-F-4-SF_5_).* Method A. Recrystallised from hot ethanol to give yellow plates (40%). Mp 118-119 °C. ¹H NMR (400 MHz, CDCl_3_) 8.37 (1H, m), 8.08 (1H, s), 7.77 (1H, m), 7.70-7.66 (1H, m). ^13^C{^1^H} NMR (100 MHz, CDCl_3_) 161, 149, 128, 123, 122, 115, 112, 111, 87. ^19^F{^1^H} NMR (376 MHz, CDCl_3_) 81-79 (1F, quint, ^2^*J*_FF_ = 152 Hz), 62 (4F, d, ^2^*J*_FF_ = 153 Hz), -107 (1F, s). LRI 1468, *m/z* (%): 298 (64), 190 (88), 171 (43), 163 (100), 151 (27), 144 (29), 139 (45), 127 (34), 124 (37), 89 (47).

*2-Fluoro-5-(pentafluorosulfanyl)benzylidenemalononitrile (2-F-5-SF_5_). Method* A. Recrystallised from hot isopropanol to give salmon-pink plates (35%). Mp 81-82 °C. ¹H NMR (400 MHz, CDCl_3_) 8.66 (1H, m), 8.08 (1H, s), 8.07-8.03 (1H, m), 7.39 (1H, m). ^13^C{^1^H} NMR (100 MHz, CDCl_3_) 162, 160, 150, 133, 127, 119, 112, 111, 87. ^19^F{^1^H} NMR (376 MHz, CDCl_3_) 81-79 (1F, quint, ^2^*J*_FF_ = 152 Hz), 63 (4F, d, ^2^*J*_FF_ = 153 Hz), -104 (1F, s). LRI 1498, *m/z* (%): 298 (83), 190 (86), 171 (29), 163 (100), 151 (30), 144 (33), 139 (48), 127 (35), 124 (43), 89 (67).

*1-Naphthylidenemalononitrile (1-Np).* Method B. Rinsed with isopropanol to give a fine bright yellow solid (86%). Mp 167-168 °C (lit. mp 166 °C [4]). ¹H NMR (400 MHz, CDCl_3_) 8.68 (1H, s), 8.30 (1H, d, *J* = 7.3 Hz), 8.14 (1H, d, *J* = 8.1 Hz), 8.00-7.96 (2H, m), 7.74-7.61 (3H, m). ^13^C{^1^H} NMR (100 MHz, CDCl_3_) 158, 135, 133, 131, 129, 128, 127.5, 127.3, 125, 122, 114, 113, 85. LRI 1984, *m/z* (%): 205 (13), 204 (84), 203 (40), 178 (26), 177 (100), 176 (32), 151 (15), 150 (20), 126 (13), 75 (18).

*Dibenz[b,f][1,4]oxazepine (CR).* Mp 72 °C. ^1^H NMR (600 MHz, CDCl_3_) 8.56 (1H, s, CH=N), 7.49 (1H, ddd, *J* = 7.8, 7.8, 1.4 Hz), 7.39 (2H, ddd, *J* = 7.8, 7.8, 1.6 Hz), 7.28-7.15 (5H, m). ^13^C{^1^H} NMR (150 MHz, CDCl_3_) 160.6 (C=N), 160.4, 152, 140, 133, 130, 129, 128, 127, 125.7, 125.1, 121, 120.

**Supplementary figure.** Antagonism of hTRPA1 activated by CS by two compounds. Oxime A-967079 had a more potent antagonistic effect than the purine HC-030031 (log_10_ EC_50_ -6.85 and -4.37 respectively).

**Supplementary table 1.** Percentage conversion of BMNs to products^a^ of addition of *N*-acetyl-L-cysteine methyl ester upon heating and cooling.

| **Analogue** | **t = 0 h**  **25 °C** | **t = 2 h**  **25 °C** | **t = 4 h**  **37 °C** | **t = 6 h**  **37 °C** | **t = 10 h**  **25 °C** | **t = 14 h**  **25 °C** | **t = 24 h**  **37 °C** |
| --- | --- | --- | --- | --- | --- | --- | --- |
| 2-F | 1.56 | 10.6 | 11.7 | 21.3 | 22.4 | 40.5 | 41.4 |
| 2-Cl ^b^ | 23.3 | 50.9 | 50.7 | 43.6 | 46.3 | 56.5 | 51.6 |
| 3-Cl | < 1.0 | 11.4 | 12.6 | 26.4 | 28.0 | 55.6 | 57.0 |
| 4-Cl | < 1.0 | 2.0 | 2.3 | 5.2 | 5.7 | 15.9 | 16.8 |
| 2-Br | < 1.0 | 6.8 | 7.7 | 16.8 | 18.2 | 55.9 ^c^ | 56.3 ^c^ |
| 2-I | < 1.0 | 7.6 | 8.7 | 16.9 | 18.4 | 38.3 | 39.8 |
| 2-NO_2_ | 2.3 | 18.1 | 21.3 | 39.3 | 42.4 | 68.8 | 69.1 |
| 2-OMe | 1.6 | 11.7 | 12.7 | 19.4 | 20.7 | 32.4 | 32.1 |
| 3-OMe | < 1.0 | 5.8 | 6.6 | 13.6 | 14.6 | 32.0 | 32.6 |
| 4-OMe | < 1.0 | < 1.0 | < 1.0 | < 1.0 | < 1.0 | 2.0 | 2.0 |
| 2-SF_5_ | 37.2 | 41.4 | 37.8 | 34.5 | 39.6 | 43.2 | 38.2 |
| CR | 38.7 | 36.8 | 28.3 | 27.6 | 37.8 | 37.3 | 28.0 |

Time *t* is the time elapsed after mixing. a. Sum of both diastereomeric products. b. CS. c. Measurement made at 72 h due to spectrometer failure.

**Supplementary table 2.** Equilibrium constants [55] for reaction of BMNs with *n-*butanethiol in aqueous phosphate buffer at pH 7 and 25 °C versus hTRPA1 responses.

| **Substituent** | **Equilibrium constant *^a^***  ***K*** × **10^-2^ (M^-1^)** | **hTRPA1 response**  **Log_10_ EC_50_ (M)** |
| --- | --- | --- |
| 2-Cl (CS) | 7.0 ± 0.6 | -8.102 |
| 3-Cl | 6.7 ± 0.1 | -6.836 |
| 4-Cl | 2.5 ± 0.2 | 67.4 * |
| 2-Br | 8.2 ± 0.6 | -7.979 |
| 3-Br | 4.6 ± 0.4 | -6.943 |
| 4-Br | 2.6 ± 0.3 | 86.6 * |
| 2-F | 4.5 ± 0.2 | -8.022 |
| 3-F | 5.5 ± 0.2 | -7.214 |
| 4-F | 1.2 ± 0.1 | 88.6 * |
| 3-OH | 1.9 ± 0.2 | -7.175 |
| 4-OH | no reaction | 1.6 * |
| 2-OMe | 1.3 ± 0.1 | -6.531 |
| 3-OMe | 2.5 ± 0.1 | -7.135 |
| 4-OMe | 0.2 ± 0.0 | 8.5 * |
| 2-OEt | 1.2 ± 0.2 | 71.1 * |
| 2-H (BMN) | 1.7 ± 0.3 | -6.847 |
| 2-Me | 0.9 ± 0.1 | -7.370 |
| 2-Et | 0.4 ± 0.0 | -6.533 |

*a.* Measured in 20% ethanol-80% pH 7 buffer (M/15 Na_3_PO_4_ (60%) and M/15 KH_2_PO_4_ (40%)). * EC_50_ could not be determined; data presented as % of maximum mean fluorescence at 3 µM, as titration curves did not reach completion. NM = not measured.
